# The effect of psychological distresses caused by COVID19 in healthcare professionals on happiness

**DOI:** 10.12669/pjms.39.1.6448

**Published:** 2023

**Authors:** Serkan Koksoy, Belkis Can

**Affiliations:** 1 Serkan Koksoy, Assistant Professor Doctor, Health Science Faculty, Mehmet Akif Ersoy University, Burdur, Turkey; 2 Belkıs Can, RN, Msc. Suleyman Demirel University, Medicine Faculty Hospital Application and Research Center, Isparta, Turkey. PhD Student. Aydın Adnan Menderes University, Department of Public Health Nursing, Faculty of Nursing, Aydin, Turkey

**Keywords:** Anxiety, COVID-19, Happiness, Psychological Distress

## Abstract

**Background and Objective:**

COVID-19 has been negatively affecting the world for a long time. This situation can trigger mental problems such as uncertainty, anxiety, stress and depression throughout the society. The aim of our study was to search the effect of the psychological distresses caused by the COVID-19 pandemic on healthcare workers (HCWs) on happiness.

**Methods:**

This study was conducted as a descriptive and cross-sectional study in a medical school hospital in Isparta-Turkey and the data for it were collected between January and March 2022. Sociodemographic form, Coronavirus Anxiety Scale (CAS), Covid-19 Related Psychological Distress Scale (CORPD) and Oxford Happiness Questionnaire Short Form (OHQ-SF) were used for the data collection.

**Results:**

A total of 326 people, including 62(19%) physicians, 127(39%) nurses, 39(12%) officers and 98(30%) others, participated in the study. Between OHQ-SF and CAS, it was found to be r^2^=0.008, f=2.76 and p=0.098. Between OHQ-SF and CORPD, it was found to be r^2^=0.11, f=3.55 and p=0.06.

**Conclusion:**

In the HCWs, it was found that CORPD was related with the variables of gender and get vaccinated, CAS was related with the variables of economic status, profession and get COVID-19, and OHQ-SF was related with economic and marital status. It was found that anxiety and psychological distress did not affect the level of happiness. It is recommended to follow up the negative effects of the pandemic, such as anxiety, fear on the future lives, professional functions, and family life of the HCW.

## INTRODUCTION

The COVID-19 disease caused by SARS Cov-2 has been affecting the world for a long time. Due to the transmission of the virus from person to person, many people were infected with this disease, people who got infected went to health institutions and therefore the burden of the healthcare system suddenly increased. In the fight against this disease, healthcare workers (HCWs) have always been at the forefront. In the early stages of the pandemic, a large number of healthcare workers were infected with COVID-19 and there were deaths resulting from COVID-19 among them.^1^ Therefore, while the HCWs were trying to provide care for people affected from the pandemic, they were also afraid of being infected with the disease.^2^ These fears and uncertainties caused the trigger of various psychological problems in health workers over time. These problems generally include anxiety, depression, stress, sleep disorders and mental health.^3^-^5^

Various scientific studies were conducted to understand the psychological states of the HCWs in the earlier stages of the pandemic.^6^ In a study conducted in Turkey, the states of the HCWs such as depression and anxiety were examined with various sociodemographic variables. According to the findings of this study, negative changes were observed in the states such as depression and anxiety caused by COVID-19.^7^ Providing healthcare service delivery is a very complex and difficult process. The fact that the states such as stress and anxiety in the HCWs providing service in this complex and difficult process are in higher levels compared to the other professions are relatively normal. However, it has been shown that the anxiety levels of the HCWs during the pandemic process are even higher than before the pandemic.^8^ According to the findings of a meta-analysis made, it has been shown that the pandemic not only triggers various psychological problems, but also negatively affects many vital activities such as sleep disorder in the HCWs.^6^

Various scientific studies have been conducted both locally and internationally in order to understand the states such as anxiety, depression, stress and fear that the HCWs experience in relation with COVID-19.^7^,^9^ Most of these studies were conducted in the earlier stages of the pandemic, when COVID-19 was not understood well, and when the vaccination was not available or common. The strict measures (quarantine, isolation, personal protective equipment, separation from the family) applied to control the pandemic during this period could negatively affect the mood of a person, as well as negatively affect the mood of the HCWs.^10^ The fear of infecting himself/herself or one of his/her family members and fighting against the density of patients in the field increased this negative situation even more.^2^ In the later stages of the pandemic, COVID-19 has been understood partially, the measures taken and the vaccines developed have become effective and the applications to health institutions have decreased. Therefore, the restrictive issues such as quarantine, isolation, mask requirement have been mitigated by the health policy makers. The scales used in the study methods used to understand the psychological problems experienced within the term passed until this mitigation period were the scales measuring general anxiety, depression, etc. In the later stages of the pandemic, the specific scales which measure states such as fear, anxiety, and suspicion directly caused by COVID-19 have been developed.^11^,^12^ The number of studies conducted on the HCWs using these specific scales is extremely limited. In addition, it is extremely important to determine with these new scales the fact that now in which level the problems such as anxiety and fear experienced in the HCWs at the earlier stages of the pandemic are. In the literature review, it was observed that the fear, anxiety and happiness parameters which are directly created by COVID-19 on the HCWs were processed together and the scientific studies using specific scales were extremely limited. For this reason, the aim of our study was to search with current scales the effect of fear and anxiety on happiness which are caused by the pandemic on the HCWs, and to show the change over time.

## METHODS

Healthcare professionals working in Isparta province were included in the research and the data were obtained from these participants. The participant criteria have been determined as having worked as a healthcare worker in a health institution for at least one year, not having any diagnosed health problems (Acute-chronic diseases, psychological disorders, etc.), having worked or being working in the units created related with COVID-19 (Intensive Care Unit, Emergency Service, Vaccination Unit, Filiation and Surveillance Teams, other services where possible and suspected COVID-19 cases are held, PCR unit (sampling) etc.).

Branches are classified as Physician (All branches), Nurse (All branches), Officer (Health Officer) and Other (Paramedic, Perfusionist, Nurse Assistant, Health Technicians etc.). Data were collected between January-March 2022 which is the period after B1.529 (Omicron) was identified.

This study was designed as a descriptive and cross-sectional study. Few of the data were collected online. Physical distance rules were applied for the data collected face to face.G*Power program was used for sample size.^13^ The G*Power program and sample determination parameters used for determining the number of samples were used in a previous study conducted in relation with COVID-19.^14^ The data entered into this program were determined as *Effect size f=0.25, α error =0.05, Power (1-β) =0.95 and Number of groups=4*, and it was calculated that the sample group should be 280 people in total. Data consists of sociodemographic data form, COVID-19 related psychological distress in healthy public (CORPD), Coronavirus Anxiety Scale (CAS), and Oxford Happiness Questionnaire Short Form (OHQ-SF). This form was created by the authors by reviewing the current literature according to the objective and design of the study.

### COVID-19 related psychological distress in healthy public (CORPD):

This scale was developed by Feng et al (2020).^11^ The Turkish adaptation of the scale was done by Ay et al (2022). The cronbach alpha value of the scale was found as 0.81, Suspicion of being inflected by COVID-19 was found as .82, and Anxiety & fear of being inflected by Covid-19 sub-dimension was found as 0.81.^15^

### Coronavirus Anxiety Scale (CAS):

This scale was originally developed by Lee (2020). The scale was found to have five items, one dimension, and the cronbach alpha value was 0.93. In addition, the cut-off score was determined as ≥9 (90% sensitivity and 85% specificity).^12^ The Turkish adaptation of the scale was made by Bicer et al. (2020). The cronbach alpha value of the scale was found to be 0.83.^16^

### Oxford Happiness Questionnaire Short Form (OHQ-SF):

The Turkish adaptation of the scale was made by Dogan and Cotok (2011) by removing a question. The cronbach alpha value of the scale was found to be 0.74.^17^

### Ethical Approval:

Ethical approval of the study was obtained from Burdur Mehmet Akif Ersoy University Non-Invasive Clinical Practices Ethical Committee (Date: 01.12.2021, No: GO 2021/418). Informed consent form was read face to face by the authors to the participants who participated in the study (“confirm button” for online data collection). Filling out the entire form by the participant has been accepted as the consent given by him/her. The Declaration of Helsinki was followed within the data collection phase.

### Statistical Analysis:

SPSS 24 package program was used in the analysis of the data. Descriptive parameters are given as number of participants n (%), mean (±) standard deviation (Mean±SD).Student t-test and f-test were used to compare groups. Statistical differences detected within the group in the ANOVA test were analyzed with the posthoc Tukey test. Multiple linear regression was performed to understand the predictive status of the independent variables for the dependent variable. Absolute value ranges (n>300) suggested in a study for Skewness and Curtosis values in normal distribution were accepted.^18^ Statistical significance was determined as 0.05.

## RESULTS

A total of 326 people, including 62(19%) physicians, 127(39%) nurses, 39(12%) officers and 98(30%) others, participated in the study. Out of which 95(74.8%) of the nurse participants were female. The mean age of HCWs were 32.3±7.6 years (min:19, max:59). The total working time of the participants in any health facility was calculated as 8±7.3 years. The study period in the COVID-19 clinics of the health facility where the data was collected was found to be 5.3±8.2 months. Among the HCWs participating in the study, 12(3.7%) people were not vaccinated for any reason.

The CORPD mean point was 36.2±10.1 (Cronbach α=0.84). The mean of fear-anxiety and doubt, which are the sub-scales of this scale, were also calculated (16.9±4.8 and 19.5±6.8, respectively). The OHQ-SF mean was calculated as 18.4±4.2 points (Cronbach α=0.77), and the CAS mean was calculated as 2.6±3.4 points (Cronbach α=0.81). The number of participants with a CAS≥9 points was found to be 19(5.8%).

Statistical differences were calculated for some of the parameters summarized in Table-I. These statistical differences were found in the parameters of gender in CORPD, economic status in CAS, and marital status as well as economic status in OHQ-SF. The mean scores of CAS, CORPD, and OHQ-SF were higher in female and married participants. In the economic situation variable, the mean of CAS was higher in the participants whose income was higher than their expenses, and the mean of CORPD was higher in those whose income was less than their expenditure, and the mean of OHQ-SF was lower in the participants whose income was less than their expenses (Table-I).

**Table-I:** The descriptive parameters of CAS, CORPD and OHQ-SF with sociodemographic variables.

Variable			n (%)	Mean±SD	t /f	p
Gender	Female	CAS	207 (63.5)	2.8±3.3	1.2	0.23
	Male		119 (36.5)	2.3±3.5		
	Female	CORPD	207 (63.5)	38.2±9.7	4	**<0.001**
	Male		119 (36.5)	33.4±11.5		
	Female	OHQ-SF	207 (63.5)	21.7±5	0.5	0.614
	Male		119 (36.5)	21.4±5.1		
Marital Status	Married	CAS	184 (56.4)	2.8±3.6	1.3	0.2
	Single		142 (43.6)	2.3±3.1		
	Married	CORPD	184 (56.4)	37.2±9.8	1.5	0.134
	Single		142 (43.6)	35.4±11.6		
	Married	OHQ-SF	184 (56.4)	22.4±4.8	3.15	**0.002**
	Single		142 (43.6)	20.6±5.1		
Economic Status	I=E	CAS	154 (47.2)	2.2±2.5	3.3	**0.038^a,a,b^**
	I<E		136 (41.7)	2.8±3.6		
	I>E		36 (11)	3.8±5.3		
	I=E	CORPD	154 (47.2)	36.7±8.8	2.08	0.127
	I<E		136 (41.7)	37.1±11.7		
	I>E		36 (11)	33.1±13.2		
	I=E	OHQ-SF	154 (47.2)	22.6±4.9	5.8	**<0.001^a,b,a^**
	I<E		136 (41.7)	20.3±5		
	I>E		36 (11)	22.6±5		

t: Student T test, f: ANOVA. I=E: Income, (I) -Expense (E) Balanced. I<E: Less Income Than Expense, I>E: More Income Than Expense, CAS: Coronavirus Anxiety Scale, CORPD: COVID-19 related psychological distress in healthy public, OHQ-SF: Oxford Happiness Questionnaire Short Form.

In Table-II, the mean of CAS was found to be high in nurses, the mean of CORPD was found to be high in physicians, and the mean of OHQ-SF was found to be high in officer. A statistical difference was found in both the profession and get COVID-19 variable of the CAS, and the CAS score was found to be high in participants with COVID-19. No statistical difference was calculated in CORPD and OHQ-SF of the same independent variables. (Table-II).

**Table-II:** The CAS, CORPD and OHQ-SF descriptive parameters of Profession and Get COVID-19.

Variable			n(%)	Mean±SD	t/f	p
Profession	CAS	Physician	62(19)	2.5±3.4	2.8	0.041^ba,a,ba,b^
		Nurse	127(39)	3.1±3.3		
		Officer	39(12)	2.9±4.6		
		Other	98(30)	1.8±2.7		
	CORPD	Physician	62(19)	38.1±13.4	0.68	0.563
		Nurse	127(39)	36±9.8		
		Officer	39(12)	36.6±10		
		Other	98(30)	35.9±10		
	OHQ-SF	Physician	62(19)	21.4±5	0.586	0.625
		Nurse	127(39)	21.8±5.2		
		Officer	39(12)	22.4±4.3		
		Other	98(30)	21.4±5		
Get COVID-19	CAS	Yes	139(42.6)	3±3.9	2.13	0.034
		No	187(57.4)	2.2±2.9		
	CORPD	Yes	139(42.6)	36.2±9.6	-0.006	0.74
		No	187(57.4)	36.6±11.38		
	OHQ-SF	Yes	139(42.6)	21.6±5	0.45	0.995
		No	187(57.4)	21.6±4.9		

t: Student T test, f: ANOVA. CAS: Coronavirus Anxiety Scale, CORPD: COVID-19 related psychological distress in healthy public, OHQ-SF: Oxford Happiness Questionnaire Short Form.

In Table-III, the means of CAS, CORPD and OHQ-SF were high in the HCWs who got all doses of vaccines developed for COVID-19 and made available by the Ministry of Health. A statistical difference was observed only in the CORPD parameter. While the mean of CAS and OHQ-SF was found to be high in those who got the inactivated (Sinovac) vaccine type, which was first used in the HCWs for vaccination, the mean of CORPD was higher in those who got mRNA Comirnaty (Biontech) + inactivated vaccine (Sinovac) type (Table-III).

**Table-III:** The CAS, CORPD and OHQ-SF descriptive parameters of Vaccine and Vaccination Type.

Variable			n(%)	Mean±SD	t\f	p
Get Vaccinated	CAS	All Doses	204(62.6)	2.7±3.4	0.58	0.562
		Missing Dose	110(33.7)	2.6±3.4		
		Not Vaccinated	12(3.7)	1.6±1.6		
	CORPD	All Doses	204(62.6)	38±10.9	7.95	<0.001^a,b,b^
		Missing Dose	110(33.7)	34.3±9.4		
		Not Vaccinated	12(3.7)	28.8±11.2		
	OHQ-SF	All Doses	204(62.6)	21.7±5.3	0.89	0.413
		Missing Dose	110(33.7)	21.6±4.6		
		Not Vaccinated	12(3.7)	19.8±2.6		
Vaccination Type	CAS	mRNA	204(62.6)	6.3±3.1	1.93	0.124
		Inactive	110(33.7)	8±4.6		
		mRNA + Inactive	12(3.7)	7.6±3.8		
		Not Vaccinated	12(3.7)	6.4±2		
	CORPD	mRNA	37(11.3)	35.6±14.6	1.66	0.177
		Inactive	80(24.5)	35.8±9.4		
		mRNA + Inactive	197(60.5)	37.2±10.1		
		Not Vaccinated	12(3.7)	30.8±11.9		
	OHQ-SF	mRNA	37(11.3)	20.7±6	1.12	0.34
		Inactive	80(24.5)	22.3±4.3		
		mRNA + Inactive	197(60.5)	21.6±5.1		
		Not Vaccinated	12(3.7)	20.7±3.6		

t: Student T test, f: ANOVA. CAS: Coronavirus Anxiety Scale, CORPD: COVID-19 related psychological distress in healthy public, OHQ-SF: Oxford Happiness Questionnaire Short Form. mRNA: Comirnaty/Biontech, Inactive: Sinovac.

In the multiple linear regression analysis made, it was determined that the CAS dependent variable was affected by the parameter of “economic status, profession and get COVID-19” (p:0.005; 0.031 and 0.019, respectively), that the CORPD dependent variable was affected by the parameter of “gender and get vaccinated” (p:<0.001 and <0.001, respectively), and OHQ-SF dependent variable was affected by the parameter of “Marital Status” (p:0.003) (Table-IV).

**Table-IV:** Prediction of sociodemographic variables on CAS, CORPD and OHQ-SF*

IV	DV	R	B**	SE	t	P	95% CI for B		Collinearity Statistics (Tolerance/VIF)
(Constant)	CAS	R:0.26 AR^2^:0.05 F:3.19 p:0.003	5.58	1.41	3.96	<0.001	2.80	8.35	
Gender			-0.45	0.38	-1.19	0.236	-1.20	0.3	0.99/1.01
Marital Status			-0.64	0.37	-1.71	0.089	-1.38	0.10	0.97/1.03
Economic Status			0.77	0.27	2.82	0.005	0.23	1.31	0.99/1.01
Profession			-0.37	0.17	-2.17	0.031	-0.70	-0.03	0.95/1.05
Get COVID-19			-0.90	0.38	-2.36	0.019	-1.66	-0.15	0.93/1.08
Get Vaccinated			-0.45	0.34	-1.33	0.183	-1.11	0.21	0.93/1.08
Vaccination Type			0.10	0.25	0.41	0.681	-0.39	0.6	0.96/1.04
(Constant)	CORPD	R:0.353 AR^2^:0.11 F:6.49 p:<0.001	52.5	4.06	12.9	<0.001	44.5	60.5	
Gender			-4.95	1.10	-4.5	<0.001	-7.1	-2.8	0.99/1.01
Marital Status			-1.81	1.08	-1.7	0.095	-3.9	0.3	0.97/1.03
Economic Status			-0.87	0.79	-1.1	0.272	-2.4	0.7	0.99/1.01
Profession			-0.27	0.49	-0.6	0.581	-1.2	0.7	0.95/1.05
Get COVID-19			-1.12	1.11	-1	0.313	-3.3	1.1	0.93/1.08
Get Vaccinated			-3.72	0.97	-3.8	<0.001	-5.6	-1.8	0.93/1.08
Vaccination Type			0.86	0.73	1.18	0.24	-0.6	2.3	0.96/1.04
(Constant)	OHQ-SF	R:0.201 AR^2^:0.02 F:1.92 p:0.066	26.1	2.12	12.3	<0.001	2.9	30.2	
Gender			-0.1	0.57	-0.2	0.862	-1.2	1	0.99/1.01
Marital Status			-1.68	0.56	-3	0.003	-2.8	-0.6	0.97/1.03
Economic Status			-0.68	0.41	-1.6	0.101	-1.5	0.1	0.99/1.01
Profession			-0.07	0.25	-0.3	0.777	-0.6	0.4	0.95/1.05
Get COVID-19			-0.15	0.58	-0.3	0.789	-1.3	1	0.93/1.08
Get Vaccinated			-0.37	0.51	-0.7	0.462	-1.4	0.6	0.93/1.08
Vaccination Type			0.06	0.38	0.15	0.879	-0.7	0.8	0.96/1.04

* Multiple Linear Regression,

** Unstandardized Coefficients, IV: Independent Variable, DV: Dependent Variable, AR^2:^ Adjusted R Square, CAS: Coronavirus Anxiety Scale, CORPD: COVID-19 related psychological distress in healthy public, OHQ-SF: Oxford Happiness Questionnaire Short Form.

The inter-scale correlation was calculated as r= +0.38 (p<0.001) between CAS and CORPD, as r= -0.09 (p=0.09) between CAS and OHQ-SF, and as r= -0.1 (p=0.06) between CORPD and OHQ-SF. In the linear regression analysis carried out by determining OHQ-SF as the dependent variable for both CAS and CORPD, it was found to be as r^2^=0.008, Durbin-Watson= 1.9, f= 2.76 and p= 0.098 in CAS. It was found to be as r^2^=0.11, Durbin-Watson=1.88, f=3.55 and p= 0.06 in CORPD.

## DISCUSSION

The participants of this study consisted of physicians, nurses, officers and other healthcare workers who have worked for a long time in the filiation, surveillance, sampling, diagnosis and treatment units of the COVID-19 pandemic. These participants are experienced people who have fought against COVID-19 on almost every front of the pandemic. In addition to working in the same health institution before the pandemic, they did not receive any psychiatric diagnosis in their past.

In our study, it was found that the psychological distress caused by COVID-19 was close to but above the scale mean value (CORPD: 36.2±10.1). The CORPD score was higher in females, married ones, those with low-income, physicians, and those who did not get COVID-19. The statistical difference was in the parameters of gender (high in females) and get vaccinated (in all doses). At the same time, while the fear-anxiety sub-dimension mean of this scale (16.9±4.8) was found to be close to and above the scale mean value (median:15p), the doubt sub-dimension mean (19.5±6.8) was found to be close to or below the scale mean value (median:21p). Various scientific studies have been carried out since the beginning of the pandemic on situations such as anxiety, depression and stress caused by the COVID-19 pandemic in the HCWs. In these studies, it has been shown that the level of mental health threatening situations such as fear, anxiety, stress and depression generally increases in the HCWs.^3^-^5^ In a study conducted during the pandemic period in Turkey, it was found that the anxiety levels of the HCWs were higher than the pre-pandemic period. The reasons for the high level of anxiety in this study were based on the high number of cases and the fact that a significant number of the HCWs (70.5%) provides care for COVID-19 patients.^8^ The scales used to understand the negative situations caused by COVID-19 are the scales which are used in the pre-pandemic period and which aim to measure situations such as anxiety, depression and stress. During the pandemic process, scales aiming to directly measure situations such as psychological disorders, fear, anxiety, and suspicion caused by COVID-19 have been developed.^11^,^12^ Unlike general fear and anxiety, these scales directly measure situations such as fear, anxiety, and suspicion related to COVID-19. The fact that the CORPD scores we obtained from our study were higher than the mean score of the scale suggests that situations such as fear-anxiety and suspicion still continue even partially. Although a significant part of the vaccination seems to have been completed and the pandemic was partially under control at the time when this study was made, the fact that psychological distress is higher in those who got all doses and in female HCWs is in line with previous scientific researches. For example, in a previous local study, anxiety was found to be higher in female HCWs than in males.^8^ It has also been shown that anxiety and fear related to COVID-19 are associated with greater vaccine acceptance.^19^ HCWs who got vaccines defined by the Ministry of Health voluntarily are those who experience the devastating effect of the pandemic on the front line. The reasons for the high level of psychological distress in female and HCWs who got all doses may be related to the service he/she works, the type of patient he/she cares for, his/her risk perception, pandemic experience and the way he/she considers the pandemic in addition to the feelings such as anxiety and fear of that person.

When the anxiety level of the participants was evaluated, the mean of CAS was higher in females, married people, those with a high income, nurses, those who got COVID-19, those who got all defined doses, and those with inactive vaccines. The statistical difference was found only in the parameters of economic status and profession. In a rapid review and meta-analysis made at the beginning of the pandemic, it has been shown that income and providing long-term care to the patient affected by COVID-19 are some of the factors that increase the risk of negative psychological outcomes.^3^ In addition, in a local study conducted in the earlier stages of the pandemic, it was determined that the anxiety levels of nurses were higher than other HCWs.^9^ Nurse and officer HCWs spend as much time with patients as doctors. A significant portion of the time spent is related to the care given to patients. In fact, not only the treatment, but also the patients’ situations such as sleeping position, nutrition and sleep are included in the services they provide. Therefore, they spend more time with the patient. Providing care for COVID-19 patients for a long time may have increased their anxiety level. In addition, the fact that the nurses (39%) participated in our study predominantly, 74.8% of whom were females, may also have affected their level of anxiety. Although there are some parameters affecting CAS, we found the prevalence of anxiety (5.8%) to be lower than the studies conducted during the pandemic period. In a meta-analysis conducted at the beginning of the pandemic process (anxiety part was compiled from 12 studies), the prevalence of anxiety was 23.2%.^6^ It can be said that the prevalence of anxiety in HCWs have decreased today compared to the previous periods of the pandemic. There may be many reasons for the fact that the CAS scores are higher than the scale mean score but the prevalence is low. Foremost among them, there may be the decrease in the number of severe patients who applied related with COVID-19, the fact that the mass vaccination is made and that the dynamics of the pandemic has been understood.

All people have been negatively affected by situations such as anxiety, depression, fear and anxiety caused by the pandemic. However, HCWs were directly exposed to the devastating impact of the pandemic. This has undoubtedly affected their mental health. With the coexistence of many negative situations, the happiness levels of HCWs were affected by these negative situations.^10^ In our study, we found that the mean of OHQ-SF (18.4±4.2) was close or below the scale mean value (median:21p), and the level of happiness made a statistical difference only in marital status and economic status. The conducted studies have shown that the COVID-19 pandemic negatively affects young adults under the age of 35, females, unemployed people and low-income people.^20^ In a study which takes the nurses as basis before the pandemic period and in which a significant part of the participants (75.2%) consisted of female healthcare workers, it was found that the level of happiness was mean and happiness was affected by various parameters.^21^ When the average age of our participants is taken into consideration, it is seen that the income parameter is consistent with the findings of Pieh et al.^20^ and their happiness level is consistent with the findings of Khorsrojerdi et al. 21

In our study, we found that both CORPD and CAS scale scores showed weak negative correlation with OHQ-SF. In a conducted study, it was found that dealing with the anxiety, stress, function and emotion focusing on them in a mass process such as COVID-19 may contribute to the prediction of happiness and that there may be a negative correlation between happiness and stress.^22^ Therefore, the negative correlation we found between happiness and psychological distress as well anxiety is supported by the literature.

CAS, CORPD and OHQ-SF findings are generally associated with various variables and practices (sociodemographic, vaccination, etc.). Despite the fact that more than two years have passed since the pandemic, the fact that the scores found are close to the scale means, or even high in some, suggest that the psychological problems in healthcare workers continue. It gives idea about the problems to be created in the long term by the problem of the request for psychological support made by the HCWs working with COVID-19 patients for a long time in a way to be twice as much as compared to the HCWs who do not provide care and treatment to COVID-19 patients.^23^ In order to prevent these negative situations, it was emphasized that awareness of the pandemic in the HCWs and the support to be provided to them are important.^24^

### Limitations

The first one of them is that the data were collected after the Omicron variant (B1.529) became predominant, and at that time the vaccination was completed in the majority of the HCWs. The second one is that the direct measurement made by two scales for the psychological distress and anxiety caused by COVID-19 may have reminded them of their emotional state in the period when the epidemic had a devastating effect. The third one of them is that the participants worked in units related to the pandemic at different periods of the pandemic may have created response bias. Finally, the data of this study is based on self-reports of the participants which can be counted among the limitations of this study.

### Clinical Implication:

The opinion that the pandemic negatively affects the psychological state of HCWs are dominant in the literature. Even, the healthcare workers who provide care for COVID-19 patients need more support compared to those not providing the said care.^23^ This situation gives information about the dimension of the existing problem they experience. This problem in HCWs are affected by various sociodemographic variables. Foremost among them, there comes the situations such as gender, economic status, occupation, being Covid-19, being vaccinated. Therefore, the sociodemographic form for the clinical studies to be conducted should be prepared carefully. These mental problems which are experienced by HCWs may increase the possibility to make mistake (malpractice, etc.) in the work they carry out. Especially in the departments where complex medical applications are made (intensive care, operating room, etc.), this situation may occur more. The situations such as the existing anxiety and fear should be measured frequently. The findings that are obtained in the said measurements will guide the awareness trainings to be given to them later.^24^ Also, the happiness levels and parameters such as fear and anxiety should be measured at the same time. The study should be made on the policies which will increase the level of happiness and decrease the level of anxiety.

## CONCLUSION

This study was conducted approximately two years after the pandemic was announced. It was determined that CORPD was associated with gender and vaccination variables, that CAS was associated with economic status, profession, and COVID-19 variables, and that OHQ-SF was associated with economic and marital status in the HCWs. We also found that anxiety and psychological distress did not affect the level of happiness. It is suggested that the effects of the long-term psychological effects of the pandemic on the profession and family life of the HcW should be followed up.

## Authors’ Contribution:

**SK:** Conceived, designed and did statistical analysis & editing of manuscript, is responsible for integrity of research.

**BC:** Did data collection and manuscript writing.

**SK and BC:** Did review and final approval of manuscript.
